# Expression and Clinical Significance of Lactate Dehydrogenase A in Colon Adenocarcinoma

**DOI:** 10.3389/fonc.2021.700795

**Published:** 2021-07-09

**Authors:** Yutong Wang, Hui Nie, Zhiming Liao, Xiaoyun He, Zhijie Xu, Jianhua Zhou, Chunlin Ou

**Affiliations:** ^1^ Department of Pathology, Xiangya Hospital, Central South University, Changsha, China; ^2^ National Clinical Research Center for Geriatric Disorders, Xiangya Hospital, Central South University, Changsha, China; ^3^ Xiangya Lung Cancer Center, Xiangya Hospital, Central South University, Changsha, China

**Keywords:** colon adenocarcinoma, LDHA, tumor metabolism, tumor immune, therapy

## Abstract

Lactate dehydrogenase A (LDHA) is an important glycolytic enzyme that promotes glycolysis and plays a crucial role in cancer cell invasion and immune infiltration. However, the relevance of LDHA in colon adenocarcinoma (COAD) remains unclear. In this study, we analyzed the correlation between the expression of LDHA and clinicopathological characteristics in COAD using immunohistochemistry analysis, and then used integrative bioinformatics analyses to further study the function and role of LDHA in COAD. We found that LDHA was highly expressed in COAD tissues compared with adjacent normal tissues, and that COAD patients with high LDHA expression levels showed poor survival. In addition, LDHA expression was closely associated with the immune infiltrating levels of CD8+ T cells, neutrophils, and dendritic cells. Our findings highlight the potential role of LDHA in the tumorigenesis and prognosis of COAD. Furthermore, our results indicate that COAD is a novel immune checkpoint in the diagnosis and treatment of COAD.

## Introduction

Colon adenocarcinoma (COAD) is a class of colorectal cancer (CRC) and the second leading cause of cancer-related deaths worldwide (1, 2). The occurrence and development of COAD results from multiple factors *in vivo* and *in vitro*, involving a series of molecules and signaling pathways (3, 4). Although substantial diagnostic and therapeutic strategies such as neoadjuvant and targeted therapies are constantly being developed, the recurrence rate of postoperative COAD is still very high, and its 5-year survival rate after distant metastasis is less than 15% (5). Therefore, there is an urgent need to identify novel biomarkers and therapeutic targets to improve the survival rate of COAD patients.

A large number of studies have shown that the COAD tumor progression can be controlled by regulating the key factors involved in glycolysis (6–8). In addition, markers of glucose metabolism (e.g., fasting glucose, C-peptide, and HbA1c) can be used as predictors of the prognostic risk of patients with COAD (9). Lactate dehydrogenase A (LDHA) is an important glycolytic molecule that promotes glycolysis by catalyzing the interconversion of pyruvate and lactate, and promotes cancer cell invasion (10, 11). Several studies have shown that LDHA expression is abnormally high in various cancers such as liver cancer, breast cancer, and oral squamous cell carcinoma, and it is closely related to the malignant progression (12–14). It was also reported that the high expression of LDHA was related to tumor volume, stage, and degree of cell differentiation and affected the disease-free survival (DFS) and overall survival (OS), which is an important indication for clinical diagnosis (15, 16). In recent years, many studies have found that LDHA is involved in lactic acid-associated tumor immune infiltration and immune escape (17, 18). However, the relationship between LDHA and the clinicopathological characteristics of patients with colon cancer as well as its role in the immune response of COAD has rarely been reported.

In this study, we analyzed the correlation between the expression of LDHA and clinicopathological characteristics in COAD using immunohistochemistry (IHC) analysis. Then, we used integrative bioinformatics analyses to further study the function and role of LDHA in COAD. In this study, we aimed to explore the role of LDHA in COAD progression and its clinical application value.

## Material and Methods

### Tissue Samples

A total of 131 paraffin-embedded, archived COAD specimens and paired adjacent normal tissue samples were obtained from Xiangya Hospital (Changsha, China). All tumor specimens with COAD after surgical resection were collected between February 2018 and May 2019. It was confirmed that none of the patients had received treatments such as chemotherapy, radiotherapy, and immunotherapy prior to resection. Clinical COAD specimens were collected with permission from the Institutional Research Ethics Committee.

### Immunohistochemistry

IHC was performed using classical biotin–streptavidin–peroxidase staining protocols (19–21). LDHA staining was detected using anti-LDHA (1:50, 3582S, Cell Signaling Technology, Danvers, Massachusetts, USA) antibody. Five high-power fields were chosen randomly, and more than 500 cells were estimated per field for the assessment of LDHA expression. The sample scores were based on both the intensity of staining and the proportion of positively stained tumor tissue: regarding the staining intensity score: 0 (negative), 1 (weak), 2 (moderate), 3 (strong); regarding the staining area score: 0 (0%), 1 (1–25%), 2 (26–50%), 3 (51–75%), and 4 (76–100%). The final score of each specimen was calculated as the intensity score × staining area score. A score ≥ 2 was considered as high LDHA expression and < 2 was considered as low LDHA expression (22). The statistical results are shown in the **Supplementary Table 1**.

### Data Collection and Bioinformatics Methods

#### GEO Database Analysis

In this study, we used three Gene Expression Omnibus (GEO) databases, containing GSE9348 (23), GSE23878 (24), and GSE17538 (25), all of which were generated using the Affymetrix Human Genome U133 Plus 2.0 platform. The GSE9348 dataset contains 70 primary CRC samples and 10 normal colon samples; GSE23878 has 35 primary CRC samples and 24 normal colon samples. Meanwhile, GSE17538 contains clinical follow-up data for 231 COAD samples, and the prognostic value of LDHA in COAD was analyzed using two indicators: OS and DFS.

#### CRN Database

CancerRNA-Seq Nexus can directly provide information on gene expression (26). We used CRN to resumptively understand the expression profile of LDHA in human COAD tissues and cell lines.

#### TIMER Database Analysis

TIMER (http://cistrome.shinyapps.io/timer) is a comprehensive analysis software for gene expression in different tumors and tumor-infiltrating immune cells (27). We analyzed the LDHA expression between tumor and adjacent normal tissues across all TCGA tumors in TIMER, and the differential expression was statistically significant.

In addition, we used TIMER to reveal the correlation between LDHA in COAD and immune cell infiltration, including CD8+ T cells, neutrophils, and dendritic cells. The gene markers of dendritic cells, CD8+ T cells, neutrophils, and tumor-associated macrophages (TAMs) were further investigated (28). The gene expression levels were displayed using log_2_ RSEM.

#### UALCAN Analysis

UALCAN (http://ualcan.path.uab.edu/index.html) is an effective online cancer data website for the validation of the identification of tumor subgroup-specific candidate biomarkers (29). We analyzed LDHA expression in COAD patients with different clinical features in UALCAN by stratifying cohorts based on type, stage, and nodal metastasis.

#### Co-Expression Network and Enrichment Analysis

cBioPortal database (http://cbioportal.org) integrates data from several databases and independent cancer research projects, which provides a visual analysis of multidimensional cancer genomics data (30). We employed this method to analyze the LDHA co-expression genes of humans in COAD. Protein interaction (PPI) analysis was performed using Cytoscape (version 3.7.2) (https://cytoscape.org) and DAVID website (https://david.ncifcrf.gov) (31). Gene ontology (GO) enrichment analysis was visualized using Metascape (http://metascape.org/) (32) and WebGestalt (www.webgestalt.org) (33).

### Statistical Analysis

The differences in means between groups were investigated using the statistical software package SPSS 23.0 with the Student’s t-test. The chi-square test and generalized linear models were used to analyze the relationship between LDHA expression and the clinicopathological characteristics of patients with COAD. The results of the correlation of LDHA expression with immune infiltration and type of immune cell markers were generated using Spearman’s correlation. *P* value < 0.05 and |Cor| > 0.39 was considered to be statistically significant.

## Results

### LDHA Is Highly Expressed in COAD

**Figure 1:**
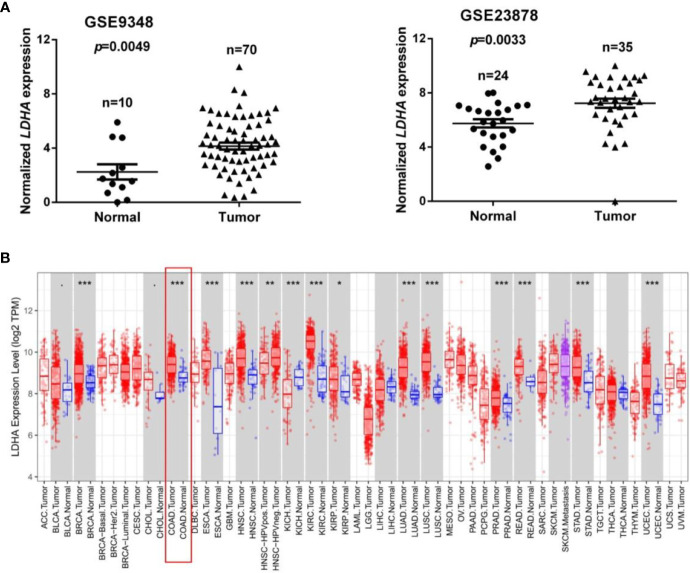
LDHA is highly expressed in COAD. **(A)** The expression level of LDHA in COAD in two GEO datasets (GSE9348: 10 normal samples and 70 COAD samples; GSE23878: 24 normal samples and 35 COAD samples). **(B)** Relative expression of LDHA in different cancers compared with normal tissues in the Oncomine database. *p < 0.05, **p < 0.01, ***p < 0.01 compared with control.

To identify dysregulated genes in COAD, two online GEO datasets (GSE9348 and GSE23878) based on the Affymetrix HG_U133 Plus 2 arrays were reanalyzed. The GSE9348 dataset contains 10 normal samples and 70 COAD samples, and the GSE23878 dataset contained 24 normal samples and 35 COAD samples. These two gene sets were, respectively, divided into a low LDHA expression group and a high LDHA expression group based on the log-rank test. Gene set enrichment analysis (GSEA) revealed that LDHA was significantly highly expressed in COAD compared with non-tumor tissues according to the GSE9348 and GSE23878 datasets (all *p* < 0.01, **Figure 1A**). Next, we analyzed LDHA mRNA expression in various human cancers using the TIMER database. The results showed that LDHA expression was significantly elevated in breast invasive carcinoma, COAD, esophageal carcinoma, head and neck cancer, kidney renal clear cell carcinoma, lung adenocarcinoma, lung squamous cell carcinoma, prostate adenocarcinoma, rectum adenocarcinoma, stomach adenocarcinoma, and uterine corpus endometrial carcinoma compared with adjacent normal tissues, and decreased in kidney chromophobe, which also established that there was a high LDHA expression in COAD (**Figure 1B**).

### Correlation of LDHA Expression With Clinicopathologic Features of COAD

**Figure 2:**
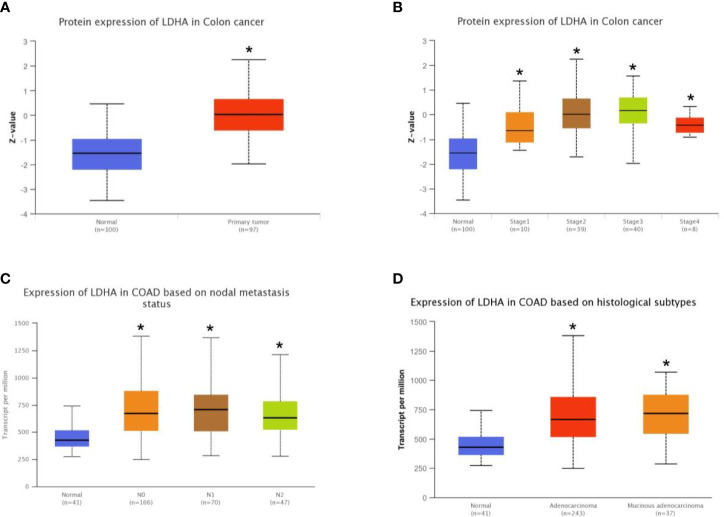
Correlation of LDHA expression and clinicopathological features of COAD was analyzed using the online database UALCAN. **(A)** Box plot shows expression of LDHA in COAD and normal patients. **(B)** Box plot shows relative expression of LDHA in normal individuals or in COAD patients in stages 1, 2, 3, or 4. **(C)** Box plot shows relative expression of LDHA in normal individuals or in COAD patients in N stages 0, 1, or 2. **(D)** Box plot shows relative expression of LDHA in normal individuals or in adenocarcinoma or mucinous adenocarcinoma COAD patients. *p < 0.05 compare with control.

To further analyze the correlation between LDHA expression and clinicopathological features of COAD, we first analyzed the protein expression of LDHA in TCGA-COAD using the UALCAN web resource and found that the protein expression of LDHA in cancer samples was higher than that in normal samples (**Figure 2A**, *p* < 0.05). Furthermore, UALCAN analysis showed that high expression of LDHA was closely related to cancer stage, nodal metastasis, and histological subtypes (**Figures 2B–D** and **Table 1**, *p* < 0.05).

**Table 1:** The expression of LDHA in TCGA COAD RNA-seq dataset were analyzed by the Cancer RNASeq Nexus.

Colon adenocarcinoma subset pair	*LDHA* (Transcript ID: uc001mok.3)		
	Average expression in cancer	Average expression in normal	Cancer *versus* Normal *P*-value
Colon adenocarcinoma–Stage I *versus* Normal (adjacent normal)	646.09	463.21	*P <*0.05
Colon adenocarcinoma–Stage II *versus* Normal (adjacent normal)	630.75	463.21	
Colon adenocarcinoma–Stage IIA *versus* Normal (adjacent normal)	669.35	463.21	
Colon adenocarcinoma–Stage IIIB *versus* Normal (adjacent normal)	779.14	463.21	
Colon adenocarcinoma–Stage IIIC *versus* Normal (adjacent normal)	642.57	463.21	

**Figure 3:**
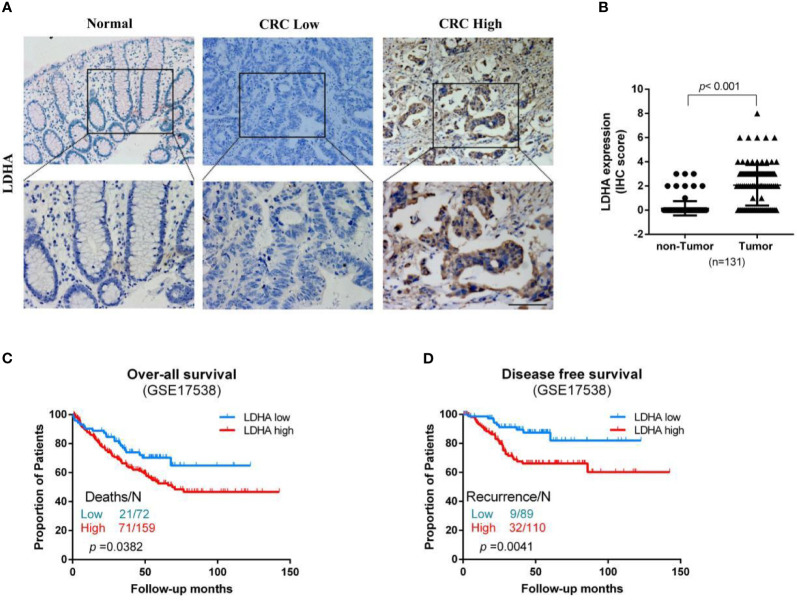
LDHA expression in COAD tissues and its correlation with prognosis. **(A)** IHC staining shows the LDHA expression in normal or COAD patients (200×). The LDHA positive tissues are stained brown. **(B)** The expression of LDHA was higher in tumor tissues of COAD compared to adjacent tissues. **(C, D)** Kaplan-Meier analysis showing overall survival (OS) and disease-free survival (DFS) curves for COAD patients with different expression levels of LDHA; statistical significance was assessed by log-rank tests.

To verify the results of the bioinformatics analyses, we assessed LDHA expression in 131 pairs of paraffin-embedded COAD tissues and adjacent normal tissues. We observed that LDHA was mainly localized in the cytoplasm, and that the expression of LDHA was significantly higher in the tumor tissues of COAD patients (**Figures 3A, B**). Next, we divided the samples into LDHA high-expression and low-expression groups according to the staining results, and the results showed that high expression of LDHA was significantly correlated with T stage, N stage, and venous invasion (**Table 2**). Furthermore, considering the low concordance in BRAF mutation status between primary and metastatic tumors (34, 35), we further analyzed the correlation between LDHA expression and BRAF status of COAD patients. However, the results showed no significant correlations between them (**Supplementary Tables 2** and **3**).

**Table 2:** Association between the expression of LDHA and clinicopathological parameters of COAD (n = 131).

Characteristics	N	LDHA		High (%)	χ2	*P*
		Low	High			
Gender					2.288	0.130
Male	73	28	45	61.64%		
Female	58	15	43	74.14%
Age (year)					0.270	0.603
≤60	75	26	49	65.33%		
>60	56	17	39	69.64%
T stage					5.674	0.017^*^
T1+T2	15	9	6	40.00%		
T3+T4	116	34	82	70.68%
N stage					4.592	0.032^*^
N0	74	30	44	59.46%		
N1+N1+N2	57	13	44	77.19%
Peripheral nerve infiltration					2.304	0.129
No	106	38	68	64.15%		
Yes	25	5	20	80.00%
Venous invasion					4.796	0.029^*^
No	90	35	55	61.11%		
Yes	41	8	33	80.49%
Tumor size (cm)					0.232	0.630
<5	86	27	59	68.60%		
≥5	45	16	29	64.44%
Differentiation degree					1.223	0.269
Poorly	22	5	17	80.95%		
Well/moderately	109	38	71	65.14%		

*p < 0.05 compared with control.

We also examined the association between LDHA expression levels and OS in the GSE17538 database using Kaplan-Meier analysis with log-rank tests. The results revealed that patients with high LDHA expression levels had lower OS (**Figure 3C**, HR=1.585; 95%CI, 1.025 to 2.451; *p*=0.0382) and DFS (**Figure 3D**, HR=2.470; 95% CI, 1.331 to 4.582; *p*=0.0041). Taken together, these data indicate that high LDHA expression is an independent risk factor for CRC patients.

### Analysis of the Co-Expression Network and Pathway Enrichment of LDHA in COAD

**Figure 4:**
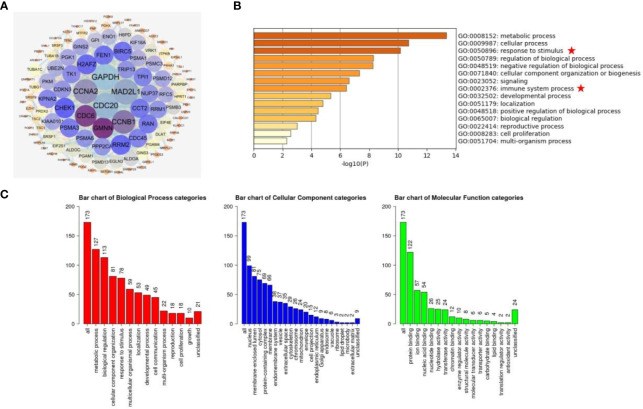
Analysis of the co-expression network and pathway enrichment of LDHA. **(A)** Using cBioPortal database and Cytoscape software, the PPI network of co-expressed gene with LDHA was constructed. **(B)** The GO enrichment pathway of LDHA co-expressed genes was analyzed on the Metascape website. **(C)** The analysis of GO biological processes, molecular functions, and cell components is from the WebGestalt website.

To analyze the biological functions of LDHA in COAD, we downloaded LDHA co-expressed genes from the cBioportal database and primary screening was carried out based on the condition of |Cor| > 0.39, *p* < 0.05. A total of 174 dysregulated genes were obtained and mapped onto a PPI network using the STRING site and Cytoscape software. From the network, we visualized that the GAPDH gene was the most relevant co-expressed gene (**Figure 4A**). GO analysis using the Metascape showed enrichment in metabolic processes, cellular processes, and responses to stimuli. Moreover, there was some correlation between these genes and pathways of immune system processes (**Figure 4B**). Furthermore, GO analysis of the 560 genes was carried out using WebGestalt, and we found that these genes were mainly enriched in metabolic processes, biological regulation, and protein binding (**Figure 4C**).

### Correlation Between LDHA Expression and Immune Infiltration in COAD

**Figure 5:**
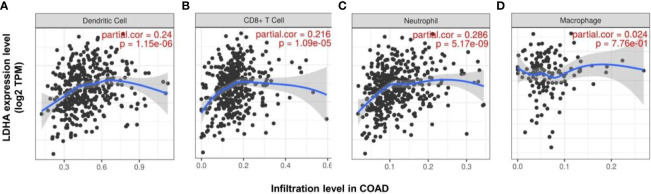
Correlation between the expression of LDHA and the level of immune infiltration in COAD. The correlation between the expression of LDHA and the immune infiltration of dendritic cells **(A)**, CD 8+ cells **(B)**, neutrophils **(C)**, and macrophages **(D)** in COAD.

As an independent prognostic factor for predicting tumor survival, immune infiltration may serve as a promising target for tumor immune-based therapy (36). Previous analysis has also revealed that the immune process is partly associated with LDHA expression. Therefore, we used TIMER to investigate the relationship between LDHA expression and immune infiltration levels. Our findings demonstrated that the expression level of LDHA was positively correlated with the infiltration level of CD8+ T cells (r = 0.216, *p* < 0.01), neutrophils (r = 0.286, *p* < 0.01), and dendritic cells (r = 0.24, *p* < 0.01) in COAD. However, LDHA expression was weakly correlated with macrophage infiltration in COAD (**Figure 5**). These results suggest the potential effect of LDHA expression on the immune response in the tumor microenvironment (TME) of COAD.

**Figure 6:**
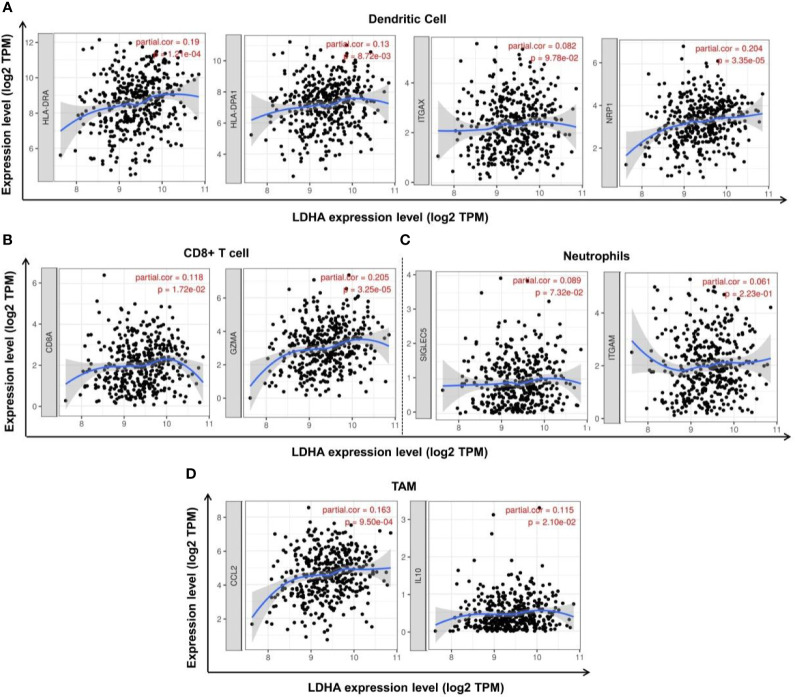
Correlation between LDHA expression and tumor markers in COAD. **(A)** Correlation between LDHA expression and dendritic cell markers HLA-DRA, HLA-DPA1, ITGAX, and NRP1. **(B)** Correlation between LDHA expression and CD8+ T cell markers CD8A and GZMA. **(C)** Correlation between LDHA expression and neutrophil cell markers SIGLEC5 and ITGAM. **(D)** Correlation between LDHA expression and TAM markers CCL2 and IL10.

To further explore the relationship between LDHA expression and various immune cells, we analyzed the types of markers of dendritic cells, CD8+ T cells, neutrophils, and TAMs in COAD using the TIMER database. We found that the dendritic cell markers HLA-DRA, HLA-DPA1, and NRP1 were positively correlated with LDHA expression (**Figure 6A**, *p* < 0.05); CD8+ T cell markers CD8A and GZMA were positively correlated with LDHA expression (**Figure 6B**, *p* < 0.05); and the TAM markers CCL2 and IL10 were positively correlated with LDHA expression (**Figure 6D**, *p* < 0.05). However, there was no significant association between LDHA expression and the neutrophil cell markers SIGLEC5 and ITGAM (**Figure 6C**, *p* > 0.05). These results further validated that LDHA is relevant to immune-infiltrating cells in COAD.

## Discussion

This study mainly analyzed the relationship between LDHA expression levels and the development of COAD. LDHA is a key enzyme involved in glycolysis. Previous studies have reported that LDHA expression is increased in many human cancers (12–14). LDHA generally promotes tumor progression by strengthening aerobic glycolysis (37). Recent studies have demonstrated that LDHA can be regulated by the upstream molecule HIF-1α and is correlated with many signaling pathways (38). In this study, we performed a comprehensive bioinformatics analysis of the relationship between LDHA expression and COAD in different public databases and found that LDHA expression was significantly upregulated in COAD tissues. Then, IHC and online database UALCAN analysis were conducted, and we found that LDHA expression is related to T stage, N stage, and venous invasion in COAD (**Table 2**), further suggesting that LDHA expression is correlated with the clinicopathological characteristics of COAD.

The gene co-expression network systematically shows the mechanism of molecular interaction and functionally corresponds to the targeted genes with specific biological processes, which broadens the way of information mining in molecular function, as well as the potential clinical application value. We constructed a co-expression analysis using cBioPortal and found that GAPDH, MAD2L1, CDC20, and CCNA2 were the most important co-expressed genes of LDHA. Interestingly, MAD2L1 was reported to be regulated by miR-30a-3p in the proliferation of gastric cancer cells (39). While derived from the 5’ end of the same stem ring, miR-30a-5p acts as a tumor suppressor in breast cancer by inhibiting the LDHA-mediated Warburg effect (37). CDC20 and CCNA2 are important genes that regulate the malignant cell cycle. They have also been reported to influence the metastasis and prognosis of COAD (40–42). Inhibitors targeting these two molecules, such as Palbociclib, TAME, and Apcin, have also been shown to inhibit tumor development (43, 44). The co-expression network indicated the crucial role of LDHA in searching for novel targets in drug effect regulation and cancer-related chemotherapy.

Interestingly, cancer immunotherapy has attracted wide attention in both basic and clinical usage (45, 46). An increasing number of studies aim to search for immune-associated inhibitors that suppress immune surveillance by decreasing the control of the TME. As in COAD immunotherapy, the combined therapy of nivolumab/lpilimumab can effectively control the progression of microsatellite instability-high COAD (47); triptolide can suppress the infiltration of macrophages in the TME and participate in the regulation of tumor cell growth and apoptosis of COAD (48); romidepsin can promote the expression of PD-L1 in COAD, and change the state of anti-tumor immune responses *in vivo* (49). Recent studies showed that LDHA may be served as a therapeutic target of cancer immunotherapy (50, 51). Oya et al. (50) found that the function of LDHA was similar to PD-L1 in the immunotherapy of non-small cell lung cancer (NSCLC). The patients with high LDHA expression levels indicated the poor efficacy of nivolumab in the treatment of NSCLC, and had shorter OS and progression free survival (PFS). Meanhwile, Qiao et al. (51) demonstrated that in the NSCLC humanized mouse model, the LDHA inhibitor Oxamate could addictively enhance the efficacy of pembrolizumab that is an anti-PD-1 immunotherapy agent. Furthermore, GO analysis revealed that LDHA expression was also correlated with the immune system process to a certain extent (**Figure 4B**), suggesting that LDHA, as a novel target, has great prospect in the field of tumor immunotherapy.

LDHA has also been shown to be correlated with diverse immune cell infiltrations in cancers. Elevated LDHA expression is associated with poor outcomes in tumor patients due to the blunt immune surveillance caused by lactic acid accumulation (17). Serganova et al. (52) showed that HIF-1α expression was inhibited in LDHA-knocked cells, accompanied by increased CD3+ and CD4+ T cell infiltration and decreased TAMS infiltration in the TME. In terms of cellular immunity, the deactivation of LDHA leads to decreased lactic acid production and neutralization of the tumor pH, which then mediates the aggregation of CD8+ T cells and NK cells, and suppresses tumor progression (53). LDHA-related lactic acid accumulation can even directly disrupt T cells and NK cells, causing tumor immune escape (17). In this study, we used TIMER to analyze the correlation between LDHA expression and immune infiltration in COAD patients. The results showed that LDHA expression was significantly positively correlated with the infiltration levels of CD8+ T cells, neutrophils, and dendritic cells. In addition, LDHA expression was positively correlated with cell markers of dendritic cells, CD8+ T cells, and TAM. These results strongly confirmed that LDHA was involved in the COAD immune response, suggesting that LDHA is a potential target for inhibiting tumor immune escape and immune tolerance. However, the specific molecular mechanism of the involvement of LDHA in tumor immune regulation is still unclear, and further research is needed to confirm this.

In conclusion, our results showed that LDHA was highly expressed in COAD tissues, and COAD patients with high LDHA expression levels showed poor survival. Meanwhile, the expression of LDHA was found to be associated with the immune-infiltrating levels of CD8+ T cells, neutrophils, and dendritic cells, thereby regulating the tumorigenesis of COAD. Our study indicates that LDHA may serve as a potential immune checkpoint for the diagnosis and treatment of COAD.

## Data Availability Statement

The original contributions presented in the study are included in the article/**Supplementary Material**. Further inquiries can be directed to the corresponding authors.

## Ethics Statement

The studies involving human participants were reviewed and approved by the Ethics Committee of the Xiangya Hospital, Central South University. The patients/participants provided their written informed consent to participate in this study.

## Author Contributions

YW and HN conducted experimental operations, sample processing, data analysis, and performed the experiments. All authors participated in writing the paper. CO and JZ conceived and designed the experiments. All authors contributed to the article and approved the submitted version.

## Funding

This study was supported by the National Natural Science Foundation of China (81903032), the China Postdoctoral Science Foundation (2020M672520), the Research Program of Hunan Health Commission, China (202103030659), the Youth Fund of Xiangya Hospital (2018Q011), the Postgraduate Scientific Research Innovation Project of Hunan Province (CX20200260), the Student Innovation Project of Central South University (2020zzts766), the Open Sharing Fund for the Large-scale Instruments and Equipments of Central South University and National Multidisciplinary Cooperative Diagnosis and Treatment Capacity Building Project for Major Diseases (Lung Cancer).

## Conflict of Interest

The authors declare that the research was conducted in the absence of any commercial or financial relationships that could be construed as a potential conflict of interest.
