# Supplementary material for: Expression and Clinical Significance of Lactate Dehydrogenase A in Colon Adenocarcinoma
Source: Front Oncol. 2021 Jul 9;11:700795. doi: 10.3389/fonc.2021.700795 (PMC8300199; doi:10.3389/fonc.2021.700795)
Supplement: Supplementary file 2 [file Table_2.docx]

**Supplementary Table 2** Association between the expression of LDHA and BRAF status of COAD (n=65)

| **Characteristics** | **N** | **LDHA** | | **High(%)** | **χ2** | ***P*** |
| --- | --- | --- | --- | --- | --- | --- |
|  |  | Low | High |  |  |  |
| BRAF status |  |  |  |  | 0.889 | 0.346 |
| Wild-type | 61 | 17 | 44 | 72.13% |  |  |
| Mutation | 4 | 2 | 2 | 50% |  |  |
