# Supplementary material for: Expression and Clinical Significance of Lactate Dehydrogenase A in Colon Adenocarcinoma
Source: Front Oncol. 2021 Jul 9;11:700795. doi: 10.3389/fonc.2021.700795 (PMC8300199; doi:10.3389/fonc.2021.700795)
Supplement: Supplementary file 3 [file Table_3.docx]

**Supplementary Table 3** Association between the expression of LDHA and BRAF status in TCGA COAD dataset (n=551)

| **Characteristics** | **N** | **LDHA** | | **High(%)** | **χ2** | ***P*** |
| --- | --- | --- | --- | --- | --- | --- |
|  |  | Low | High |  |  |  |
| BRAF status |  |  |  |  | 0.732 | 0.606 |
| Wild-type | 515 | 292 | 223 | 43.30% |  |  |
| Mutation | 36 | 22 | 14 | 38.89% |  |  |
